# Inhibitor of H3K27 demethylase JMJD3/UTX GSK-J4 is a potential therapeutic option for castration resistant prostate cancer

**DOI:** 10.18632/oncotarget.19100

**Published:** 2017-07-08

**Authors:** Viacheslav M. Morozov, Ying Li, Matthew M. Clowers, Alexander M. Ishov

**Affiliations:** ^1^ Department of Anatomy and Cell Biology and Health Cancer Center, University of Florida College of Medicine, Gainesville, FL 32610, USA; ^2^ Department of Cellular and Molecular Biology, Florida Agricultural and Mechanical University, Tallahassee, FL 32307, USA; ^3^ IDP Graduate Program, University of Florida College of Medicine, Gainesville, FL 32610, USA

**Keywords:** castration resistant prostate cancer, JMJD3/UTX, H3K27Me2/3, Cabazitaxel, GSK-J4

## Abstract

Androgen receptor (AR) mediates initiation and progression of prostate cancer (PCa); AR-driven transcription is activated by binding of androgens to the ligand-binding domain (LBD) of AR. Androgen ablation therapy offers only a temporary relief of locally advanced and metastatic PCa, and the disease eventually recurs as a lethal castration-resistant PCa (CRPC) as there is no effective treatment for CRPC patients. Thus, it is critical to identify novel targeted and combinatorial regimens for clinical management of CRPC.

Reduction of the repressive epigenetic modification H3K27me2/3 correlates with PCa aggressiveness, while corresponding demethylases JMJD3/UTX are overexpressed in PCa. We found that JMJD3/UTX inhibitor GSK-J4 reduced more efficiently proliferation of AR-ΔLBD cells (CRPC model) compared with isogenic AR-WT cells. Inhibition of JMJD3/UTX protects demethylation of H3K27Me2/3, thus reducing levels of H3k27Me1. We observed that the reduction dynamics of H3K27Me1 was faster and achieved at lower inhibitor concentrations in AR-ΔLBD cells, suggesting that inhibition of JMJD3/UTX diminished proliferation of these cells by hindering AR-driven transcription. In addition, we observed synergy between GSK-J4 and Cabazitaxel, a taxane derivative that is approved for CRPC treatment. Collectively, our results point at the H3K27 demethylation pathway as a new potential therapeutic target in CRPC patients.

## INTRODUCTION

Prostate cancer (PCa) is the most common non-cutaneous neoplasm in the Western male population and is the second leading cause of cancer mortality in American men [1]. About 1 out of 7 men would be diagnosed with prostate cancer through their lives and PCa is the second most lethal cancer for American men with estimation of 161,360 new cases and 26,730 mortalities in 2017 (American Cancer Society, 2017). Pathologic growth of the prostate is controlled mainly by steroid androgens, thus locally advanced and metastatic diseases are treated with androgen ablation therapy that aims to suppress testosterone production with hormone agonists, or block androgen receptor (AR) activation with anti-androgens that all bind the AR ligand-binding domain (LBD) [2]. Although these therapies are initially effective in about 90% of patients, a major limitation is that they offer only a temporary relief and the disease eventually recurs with a lethal outcome. PCa that relapsed after hormonal therapies is the major cause of disease lethality and is referred to as castration-resistant PCa (CRPC) [3].

Mechanisms involved in the transition of PCa from androgen-dependent to CRPC are not well established and their identification presents an opportunity to improve disease diagnosis and outcome. The AR is overexpressed in ∼30% of CRPC compared to untreated primary PCa or normal prostate tissue, thus abiraterone and enzalutamide are approved for CRPC treatment [4]. In addition to overexpression, several AR activating point mutations have been detected in human PCa [5]; these gain-of-function point and deletion mutations in AR are implicated in the development of CRPC and in therapy resistance [4]. AR mutants can be activated by non-androgens or even anti-androgens. For example, acquired resistance to the second-generation anti-androgen enzalutamide results from a missense (F876L) mutation in the LBD of AR [6]. Another mechanism of CRPC insensitivity to anti-androgens is the expression of AR deletion mutants that lack LBD [7] (ARΔLBD). Clinical data and animal models both confirm that expression of ARΔLBD determines resistance to anti-androgens in CRPC [7].

AR-induced genes are substantially regulated by epigenetic modifications of chromatin that includes post-translational histones modifications. Identification of AR epigenetic co-activators should provide targeted and combinatorial regimens for clinical management of CRPC [8]. H3K27 two-/three-methylation is a transcription repressive marker, which shows specific pattern in PCa. Study by Pellahuru et al identified correlation of reduced H3K27me3 and PCa aggressiveness [9]; moreover, H3K27me3 is reduced in tumors with metastatic PCa compared to normal PCa [10]. Demethylases JMJD3 and UTX (KDM6B and KDM6A) specifically demethylate H3K27me3/me2, resulting in transcription activation [11]. Both these enzymes were found drastically overexpressed in PCa tissues and those with higher JMJD3 level were associated with more advanced disease stage [11], indicating that PCa progression is related to the promoted demethylation of H3K27me3/me2. Thus, inhibition of JMJD3/UTX may reduce AR-driven transcription and proliferation of cancer cells in PCa and CRPC.

In this study, we investigated JMJD3/UTX inhibition by small molecule inhibitor GSK-J4 as a tool to reduce proliferation of PCa cells. GSK-J4 is a selective JMJD3/UTX inhibitor that triggers the enrichment of H3K27me3 in cell line models [12] and is suggested as a potential treatment option in the acute lymphoblastic leukaemia [13] and brainstem glioma [14]. We observed that GSK-J4 reduces proliferation of PCa cells with AR-WT and CRPC cells that express ARΔLBD. Importantly, ED50 in the last cells was three to four folds lower compared to cells expressing AR-WT, indicating elevated cytostatic/cytotoxic activity of GSK-J4 in CRPC cells. Our observations suggest that H3K27 demethylation pathway is a new potential treatment target in CRPC patients.

## RESULTS

### GSK-J4 effectively represses PCa cells proliferation

AR deletion mutants that lack LBD (ARΔLBD) were identified in several CRPC cell lines; however, in these cells ARΔLBD’s are co-expressed with AR-WT [15] that makes it difficult to study function of ARΔLBD. To circumvent this problem, the TALEN-based genome editing method was used to create cell lines R1-D567 and R1-I567, expressing AR with deleted/inverted for exons 5-7, correspondingly [16]; these cell lines derived from PCa CWR-R1 sub-clone R1-AD1 expressing WT AR [16].

**Figure 1:**
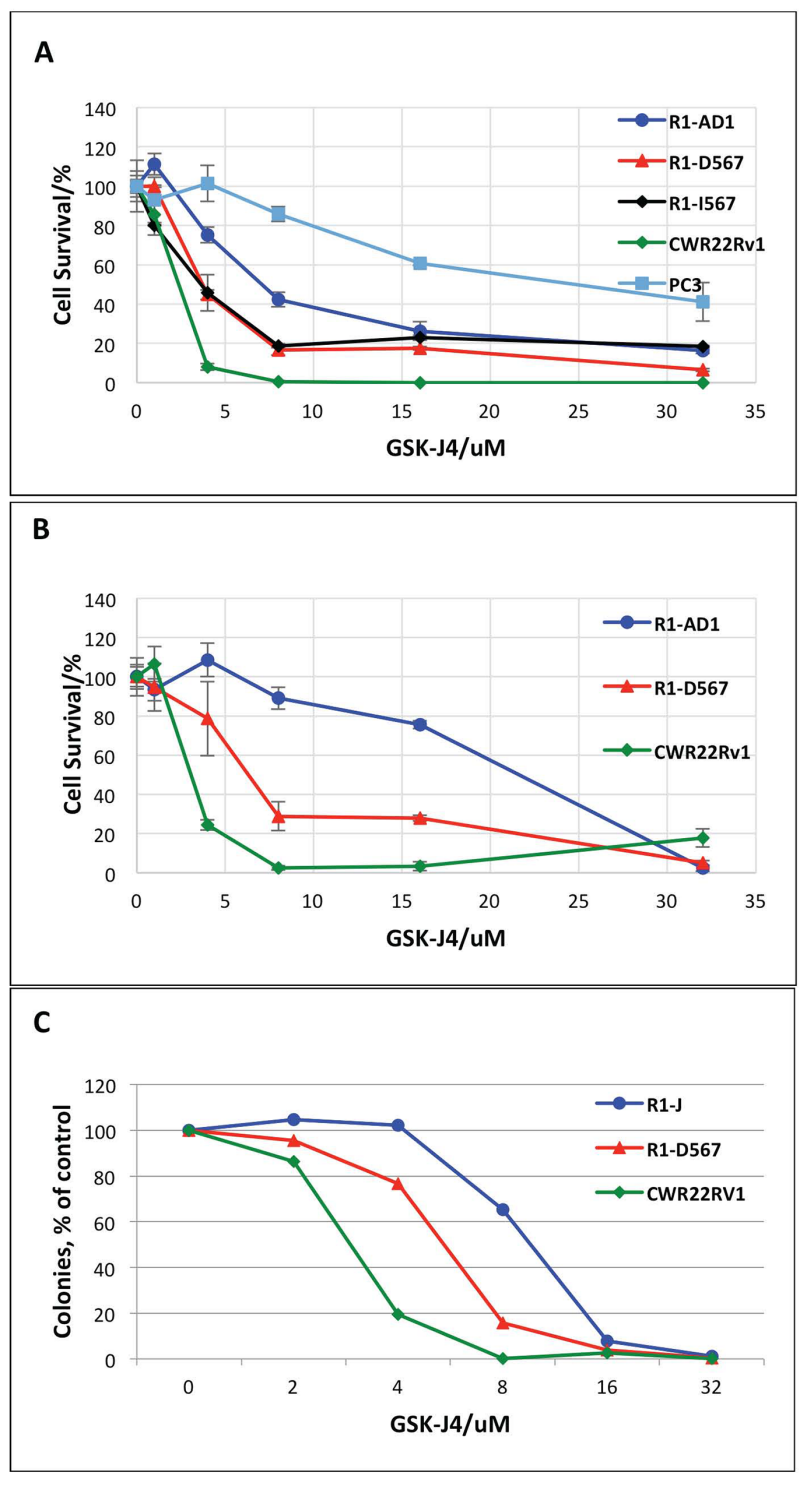
GSK-J4 treatment represses PCa cells proliferation **(A)** R1-AD1 (expressing AR-WT), R1-D567 and R1-I567 (expressing AR-ΔLBD), CWR22Rv-1 (expressing both AR-WT and AR-ΔLBD), and PC3 cells were treated with GSK-J4 at indicated concentrations for 72 h. Percent of cells that survive treatment with GSK-J4 is shown. **(B)** Proliferation of GSK-J4 treated R1-AD1, R1-D567 and CWR22Rv-1 cells were evaluated by the Alamar Blue Assay. **(C)** Colony formation assay on R1-AD1, R1-D567 and CWR22Rv-1 cell lines: cells were treated with indicated concentrations of GSK-J4 for 72 h, and then re-plated in media without drug for colony formation. Number of colonies after treatment relatively to number colonies with no treatment (100%) is shown.

The jumonji (JMJ) family of histone demethylases is an essential component of epigenetic transcription regulating machinery that is involved in numerous pathological processes, including cancer initiation and progression [17]. Correlation between reduced levels of H3K27Me3, elevation of JMJD3/UTX and aggressiveness of PCa suggests function of JMJ histone demethylases in PCa etiology; thus, inhibition of these enzymes can be considered for PCa treatment. Recently, the small molecule inhibitor of JMJD3/UTX, GSK-J4, was developed and characterized in reduction of the pro-inflammatory macrophage response [12]. We tested the effect of JMJD3/UTX demethylase inhibitor GSK-J4 on five prostate cancer cell lines: R1-AD1 (AR-WT), R1-D567 and R1-I567 (ARΔLBD), CWR22Rv-1 (CRPC cell line that expresses both AR WT and AR with deletion), and PC3 (low levels of AR expression) by the direct cell count after 72h of treatment. Cell survival curve showed reduced proliferation for all cell lines, indicating cytostatic and/or cytotoxic effect of GSK-J4 at PC cells (Figure 1A). In addition, we observed differential concentration-dependent response: CWR22Rv-1 was the most sensitive to treatment with 50% reduction of cell proliferation (50% of effective dose, ED50) ∼3 μM. For R1-D567 and R1-I567 ED50 was ∼4 μM, while the same cell survival level was observed at doses of ∼8 μM for R1-AD1. The most resistant cell line was PC3 with ED50 ∼24 μM.

Next, we continued our studies on three cell lines: CWR22Rv-1, R1-D567 and R1-AD1. The effect of GSK-J4 to these three cell lines was confirmed by cell viability test using Alamar® Blue assay (Figure 1B), which shows cell survival similar to the cell counting data (Figure 1A). The ED50 of GSK-J4 acquired from the survival curve (Figure 1B) was 3.5 μM for CWR22Rv-1, 6.3 μM for R1-D567 and 21.5 μM for R1-AD1. Observed difference in ED50 between Figure 1A and 1B can be attributed to the different methods of analysis. In addition, we performed colony formation assay on these three cell lines; cells were pre-treated with different concentrations of GSK-J4 for 72 h, and re-plated in media without drug for the colony formation (Figure 1C). CRPC cell lines CWR22Rv-1 and R1-D567 formed reduced number of colonies after GSK-J4 treatment compared with R1-AD1, confirming that the CRPC cell lines are more sensitive to GSK-J4 treatment. We observed the same tendency in cell survival: CWR22Rv-1 remained the most sensitive to treatment with 50% reduction of colony formation (50% of effective dose, ED50) ∼3 μM. For R1-D567 ED50 was ∼ 6 μM, and ∼12 μM for R1-AD1. We also tested cellular response to treatment with 6μM GSK-J4 for 72 h by microscopy (Supplementary Figure 1). While changes in R1-AD1 were minimal, treatment reduced proliferation of R1-D567 and, to the higher extend, of CWR22Rv-1 cells. It is unlikely that the difference between cell lines response to GSK-J4 can be explained by the difference in the total enzymatic activity, as levels of JMJD3 and UTX proteins was similar between these cell lines (Supplementary Figure 2). Altogether, results indicate that GSK-J4 is effective to reduce proliferation in all tested PCa cell lines and the CRPC cell lines are more sensitive to the treatment than AR-WT expressing PCa cell lines.

### Proliferation dynamics of PC cells treated with GSK-J4

**Figure 2:**
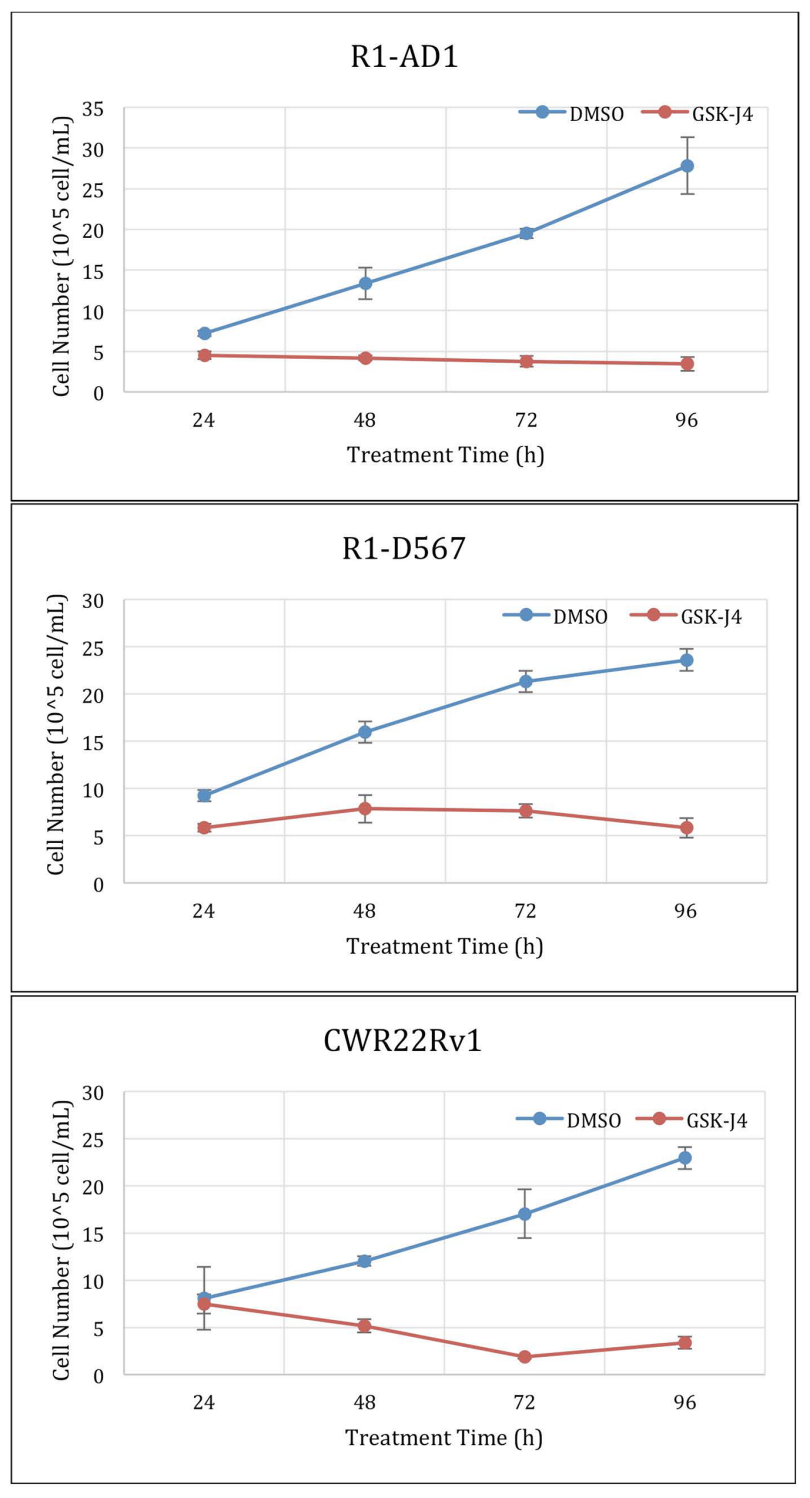
GSK-J4 time course treatment Cells were treated with the ED50 of GSK-J4: 4μM for CWR22Rv-1 (expressing both AR-WT and ΔLBD, top panel), 6μM for R1-D567 (expressing AR-ΔLBD, middle panel), and 20μM for R1-AD1 (expressing AR-WT, bottom panel). Proliferation dynamics was evaluated at 24, 48, 72 and 96 h by cell number counting.

At the next step, we performed time-course cell viability test using treatment with ED50 determined in Figure 1B for the three studied cell lines respectively. Treatment was done for 24 h, 48 h, 72 h and 96 h. The results in Figure 2 show that pre-determined EC50 doses are effective in blocking proliferation. The time-course treatment also shows that GSK-J4 affects cell lines differently. EC50 treatment of R1-AD1 cells has a minor cytotoxic effect, EC50 treatment of R1-D567 is mostly cytostatic during first 72 h, and at 96 h has a minor cytotoxic effect. EC50 treatment of CWR22Rv-1 is cytotoxic during the first 72 h. Proliferation of the first two cell lines was affected already during the first 24 h of treatment, while effect on CWR22Rv-1 is minimal during that time. The difference in the time course dynamics may indicate differential response to GSK-J4 treatment between cell lines that may combine block of cell proliferation and induction of cell death.

### GSK-J4 treatment generates persistent potency

**Figure 3:**
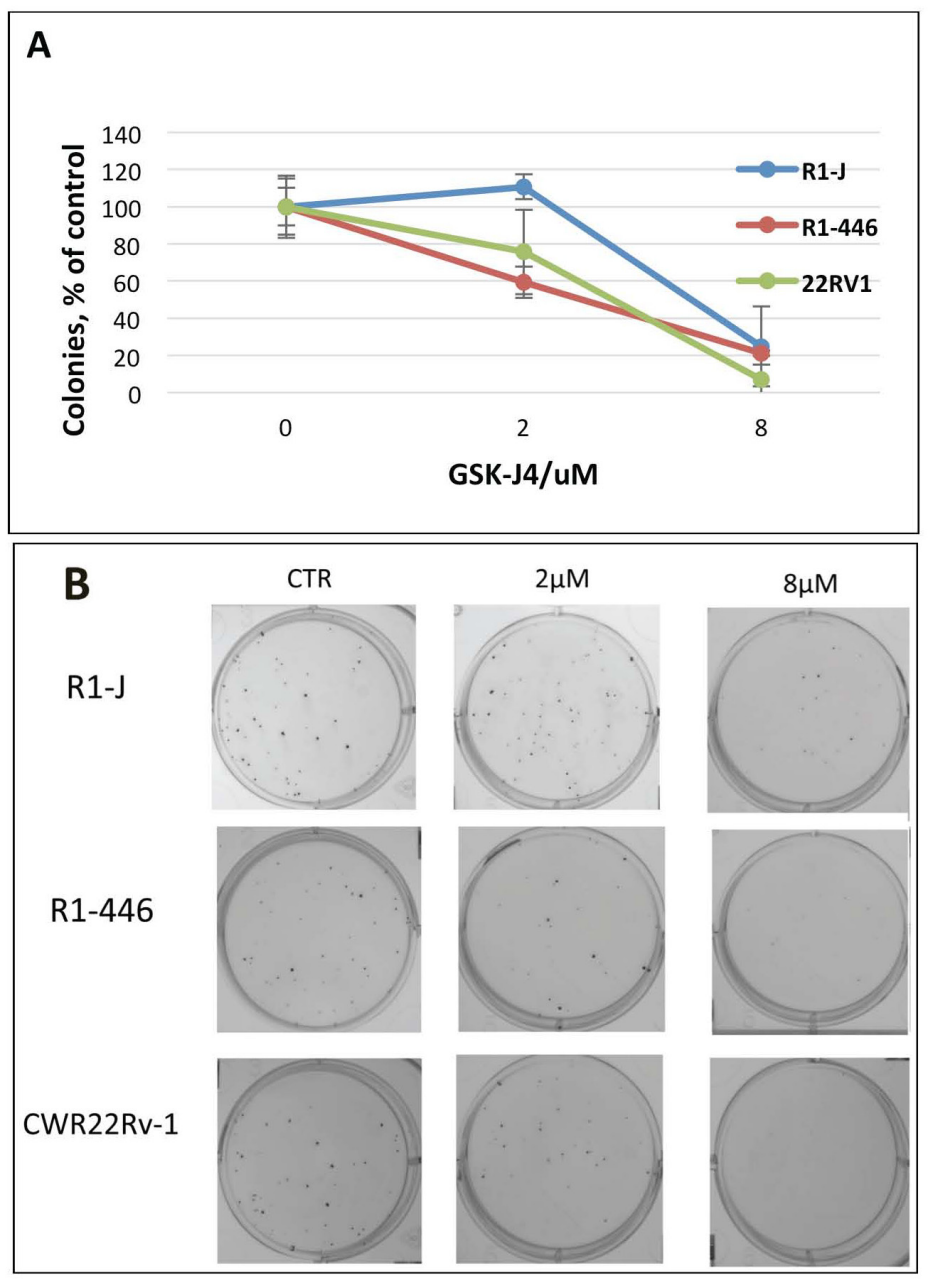
Analysis of GSK-J4 post-treatment effect by colony formation assay Cells were treated with indicated concentrations of GSK-J4; after 72 h of treatment, 200 viable cells were plated for colony formation. **(A)** Relative number of colonies (% of number colonies without treatment); **(B)** representative pictures of colonies.

In order to test for the post-treatment effect of JMJD3/UTX demethylase inhibition, we next performed the colony formation assay with the GSK-J4 pre-treated cells. In these experimental settings, cells were pre-treated for 72 h with different concentrations of inhibitor, and next 200 survived cells (determined by Trypan Blue exclusion) were re-plated for the colony formation without drug. The result in Figure 3 shows the colonies formed by survived cells retrieved after 72 h GSK-J4 treatment from all tested cell lines. In pre-treated cells, the number of formed colonies was much less then number of the plated cells, suggesting post-treatment effect of GSK-J4. Within each cell line, the number of the formed colonies negatively correlated with the applied GSK-J4 dose (Figure 3), suggesting that treatment by GSK-J4 has the long-time post-treatment effect on cell proliferation.

### GSK-J4 treatment regulates H3K27 methylation levels

**Figure 4:**
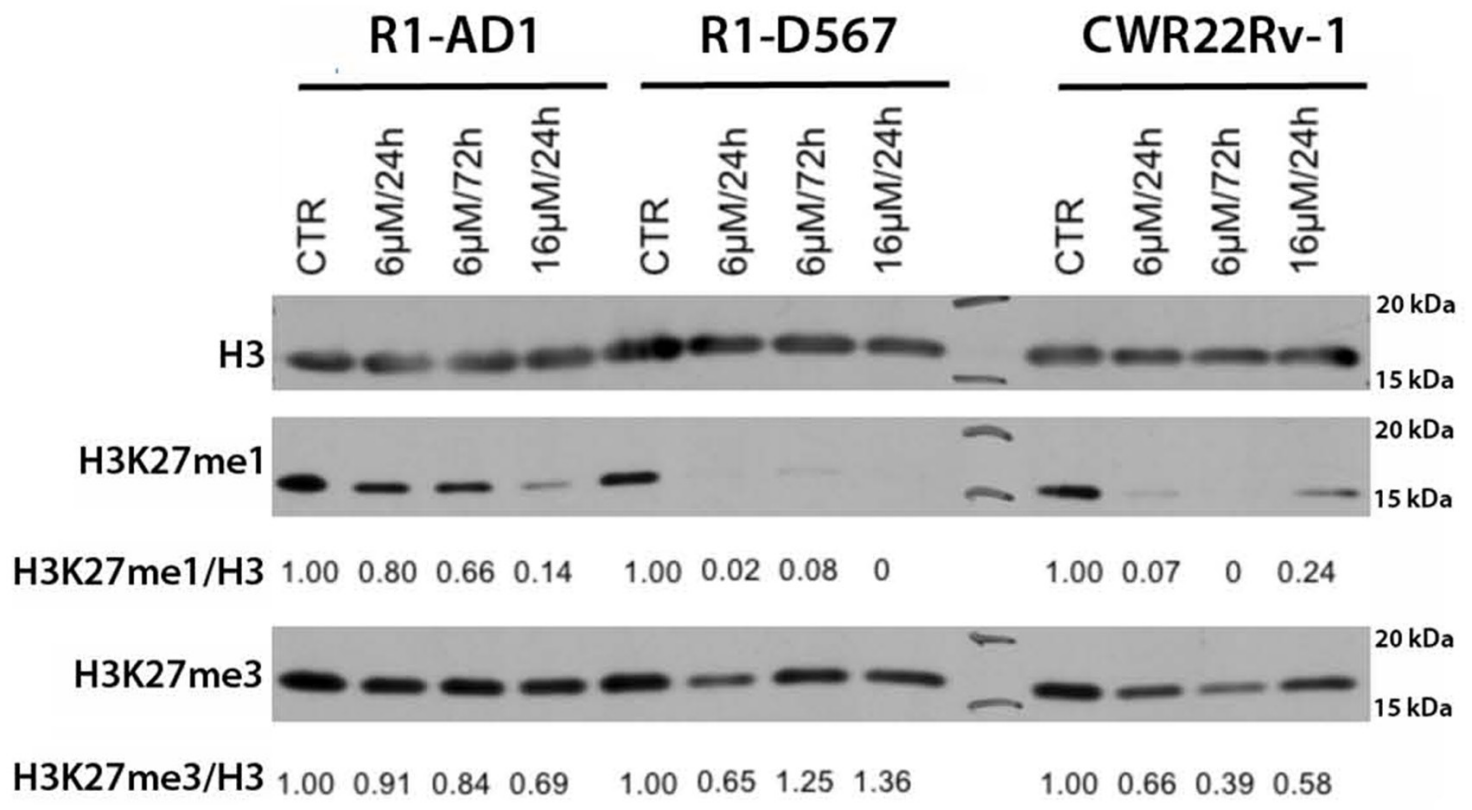
GSK-J4 treatment affects levels of H3K27 methylation Cells were treated with 6μM and 16 μM of GSK-J4 for 24 or 72 h; levels of H3K27me1 and H3K27me3 were evaluated by Western blot analysis and quantified relative to the total levels of histone H3. The reduction dynamics of H3k27me1 was faster and achieved at lower inhibitor concentrations in cells expressing AR-ΔLBD compared with AR-WT expressing cells.

Inhibition of JMJD3/UTX demethylation activity supposes to shift a ratio between H3K27 mono-, di-, and trimethylation, specifically reducing the first and accumulating the other two epigenetic modifications. In fact, reduction of H3K27 monomethylation was reported in the study characterizing activity of GSK-J4 as an inhibitor of JMJD3/UTX in acute lymphoblastic leukemia [13]. Thus, we tested dynamics of these epigenetic markers in all three cell lines at two different concentrations of inhibitor and at two different time points of treatment. Accumulation of H3K27me3 and the decrease of H3K27me1 were expected after the inhibition of demethylases JMJD3/UTX with GSK-J4. Total levels of histone H3 was used to normalize H3K27me3 and H3K27me1 level in this assay. The result shows that H3K27me1 drastically decreased after GSK-J4 treatment in all cell lines tested (Figure 4). We observed that the reduction dynamics of H3K27me1 was faster and achieved at lower inhibitor concentrations in both CRPC cell lines compared with R1-AD1, that correlated with the increased sensitivity of these CRPC cells to GSK-J4 treatment. Surprisingly, we observed minor accumulation H3K27me3 in R1-D567 cells, and no accumulation was observed in R1-AD1 or CWR22Rv-1 cells (Figure 4).

### Analysis of GSK-J4 and Cabazitaxel combined treatment

Taxanes (paclitaxel and docetaxel) are powerful and commonly used anti-neoplastic agents for the treatment of several malignancies [18, 19]. Cabazitaxel, a taxane derivative, is one of the few therapeutic options recently approved for CRPC treatment [20]. Despite identical target (hyper-polymerization of microtubules), Cabazitaxel has clinical advantage compared to docetaxel [21] suggesting additional drug activity [22]. Thus, it was tempting to test whether treatment that combined GSK-J4 and Cabazitaxel can be more effective compared to the single-agent treatments. First, we tested the effect of Cabazitaxel treatment (72 h) by cell viability test using Alamar® Blue assay. Cabazitaxel ED50 was ∼4nM (Supplementary Figure 3).

**Figure 5:**
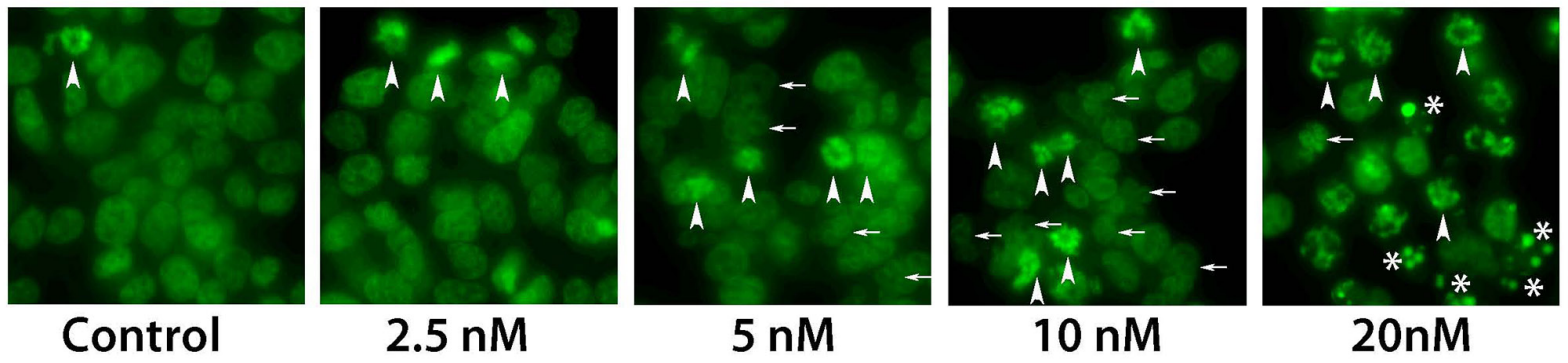
Microscopy analysis of Cabazitaxel treatment Analysis of mitotic block with different concentrations of Cabazitaxel. Cells were treated with indicated concentrations of Cabazitaxel for 24 h, and DNA was stained with the cell-permeable Hoechst 33342 followed by cell fixation and microscopy analysis. With increased concentrations of Cabazitaxel, in addition to mitotic block (arrowheads), number of micronucleated cells increased (indicative for mitotic catastrophe, small arrows). Further increase concentrations of Cabazitaxel induced highly condensed DNA morphology that is characteristic for apoptotic cells (*).

Taxanes hyper-polymerize microtubules and trigger cell death, mainly through a mitotic arrest following the activation of the spindle assembly checkpoint (SAC) [23]. Cells treated with taxanes eventually exit from this mitotic block as micronucleated cells and next die by the mitotic catastrophe [24]. Thus, similar to taxanes, the main expected mechanism of Cabazitaxel action, at least at low concentrations, can be the activation of mitotic block. We performed microscopy studies to evaluate consequences of Cabazitaxel treatment in R1-D567 cells. Cells were treated with indicated concentrations of Cabazitaxel for 24 h, and then DNA was stained with the cell-permeable Hoechst 33342 followed by cell fixation and microscopy analysis. We used cell morphology evaluation to discriminate between interphase, mitosis, micronuclei and apoptosis, as described previously [25]; representative images are presented in Figure 5. As expected, accumulation of mitotic cells was observed after treatment with Cabazitaxel. Higher concentrations, in addition to mitotic block, resulted in increase of micronucleated cells, indicating that PCa cells exit from Cabazitaxel-induced mitotic block *via* mitotic catastrophe, similar to other taxanes [25]. With further increased concentrations of Cabazitaxel, the accumulation of cells with highly condensed DNA morphology, which is characteristic for apoptotic cells, pointed to the activation of apoptosis directly from mitotic block, as described previously [26].

**Figure 6:**
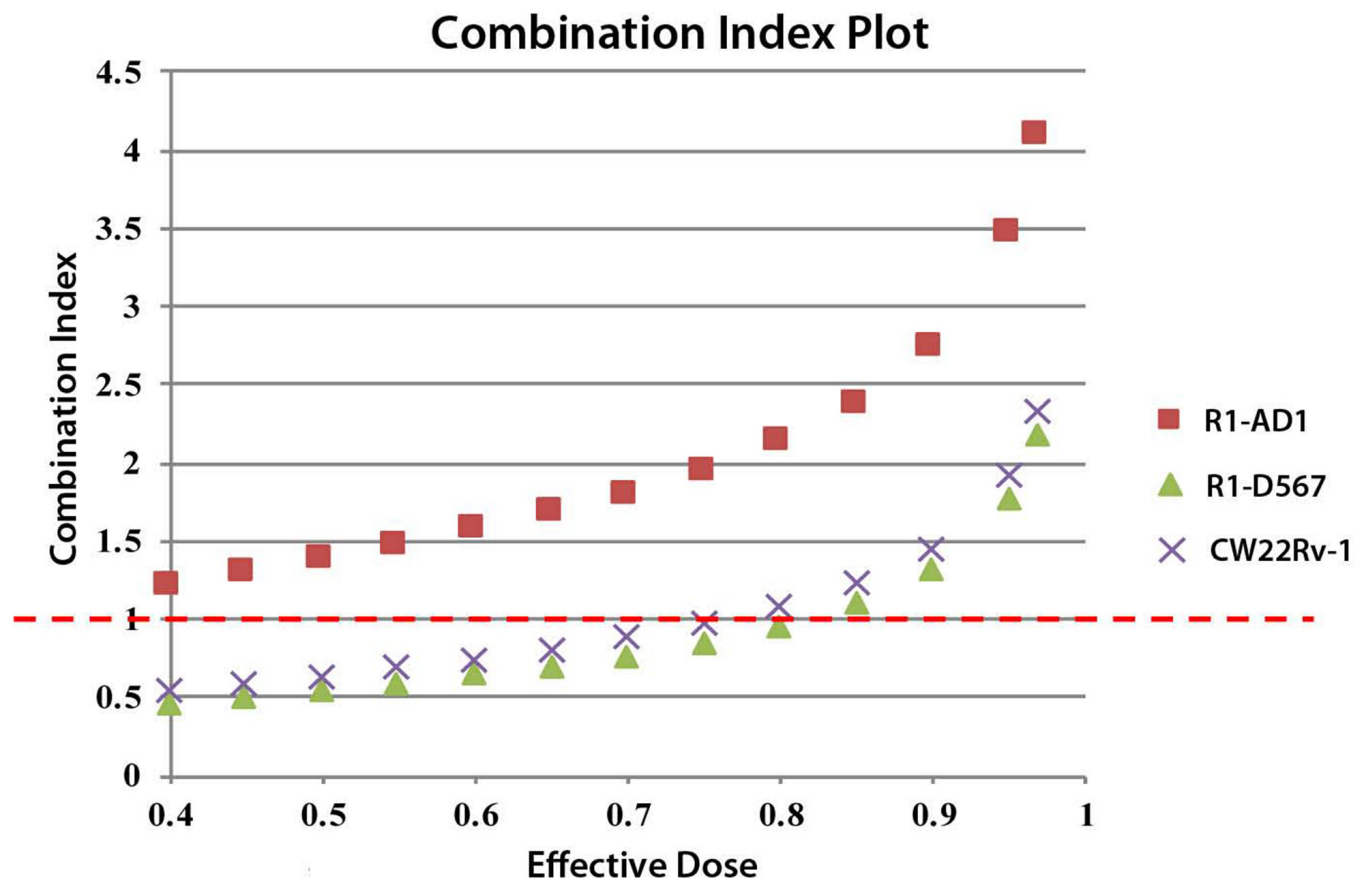
Synergistic analysis of GSK-J4 and Cabazitaxel treatment Synergistic analysis based on Chou and Talalay’s model was performed to get the combination index (CI) using CompuSyn analysis software. CI less than 1 indicates the synergism of the multi-drug treatment, while CI =1 means additive effects and CI >1 indicates antagonism.

Next, we tested effectiveness of treatment that combined GSK-J4 and Cabazitaxel on three cell lines. Synergistic analysis based on Chou and Talalay’s model was performed to get the combination index (CI). CI less than 1 indicates the synergism of the combined treatment, while CI =1 means additive effects and CI >1 indicates antagonism. No beneficial effect was found in combined treatment for R1-AD1. On the other hand, for castration resistant cell lines R1-D567 and CWR22Rv-1, elevated inhibition of proliferation was observed in combined treatment (Figure 6). The ED50 was calculated using CompuSyn (Table 1). The synergistic data shows that combined treatment of GSK-J4 and Cabazitaxel generated drug synergy at low doses in the CRPC cell lines R1-D567 and CWR22Rv-1.

**Table 1:** Effective dose 50 (ED50)

Effective dose 50 (ED50)			
	GSK-J4 (μM)	Cabazitaxel (nM)	Combined (1000:1)
**R1-AD1**	4.06	3.68	5.62
**R1-D567**	3.36	3.15	1.85
**CWR22Rv1**	1.68	2.83	1.09

The ED50 for Cabazitaxel, GSK-J4, or combined doses of Cabazitaxel and GSK-J were calculated using CompuSyn.

## DISCUSSION

The comprehensive and effective therapeutic options are limited for CRPC. The basic philosophy of CRPC treatment is vague and the medical practice mostly follows the protocols for metastatic prostate cancer treatment. The most common practice for CRPC treatment includes mitoxantrone-steroid or docetaxel-presnisone combinations as chemotherapy approaches and abiraterone and enzalutamide as hormone control approaches for first-line treatment, followed by the second-line therapies such as Cabazitaxel or radium-233 dichloride [27]. The existing treatment options have overall survival advantage range from 15 to 26 months [27]. The therapeutic efficacy greatly varies because of individual difference of patients and the subtle treatment sequencing. Yet, no systematic treatment standard for CRPC is developed. New treatment explorations that are promising for phase II clinical trial include enzymatic inhibitors such as cabozantinib, inhibitor of c-Met and VEGFR2 [28], abiraterone, enzalutamide, orteronel (TAK-700) to target androgen signaling [29], immunotherapy using Ipilumimab [30] and PROSTVAC-VF to target PSA [31]. Thus, there are several promising therapeutic directions, yet none have developed into clinical practice. Identification of effective CRPC treatment options remains the most important and challenging gap in PCa management.

Components of epigenetic machinery were recently considered as a novel class of promising targets in cancer therapeutics. In this direction, Duan et al reported an epigenetic-targeting treatment alternative for PCa using JMJD2 inhibitor to stabilize H3K9 methylation level, thus repressing AR-driven transcription [32]. Unfortunately, this study did not show any promising results for CRPC treatment, and emphasized necessity of further studies in direction of epigenetic players as potential targets in CRPC.

**Figure 7:**
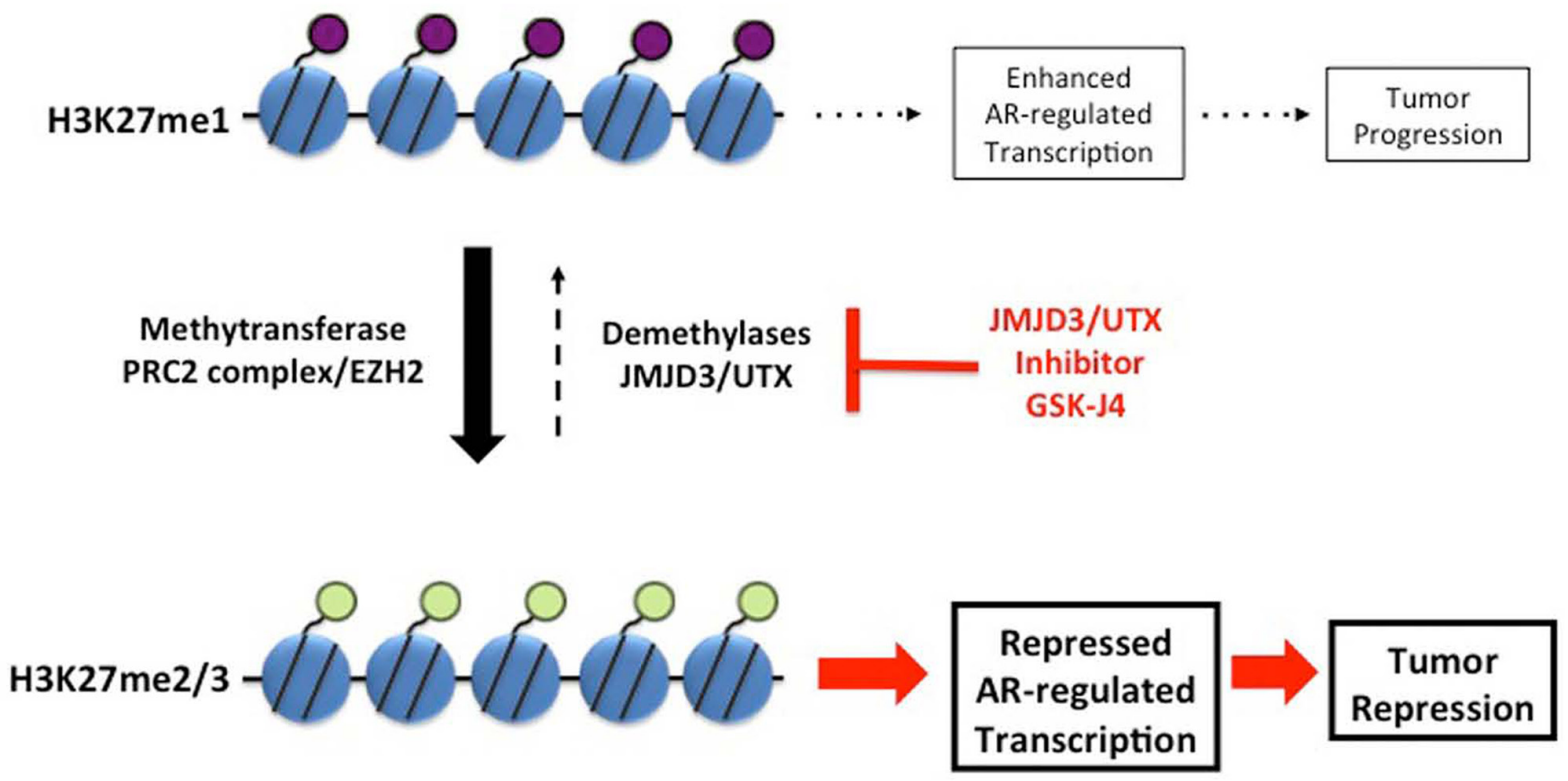
Model Balance between transcription-repressive H3K27me2/3 and transcription-permissiveH3K27me1 modifications is maintained by methyltransferase activity of PRC2 complex and demethylation activity of JMJD3/UTX. Inhibition of JMJD3/UTX by GSK-J4 shifts this balance towards transcription-repressive H3K27me2/3 modification. Protection of H3K27me2/3 modification represses AR-driven transcription, thus reducing proliferation of PCa and CRPC cells.

JMJD3/UTX are overexpressed [11] whereas levels of H3K27me2/3 are reduced in the aggressive PCa [9, 10]. Based on these data and on the postulated function of H3K27 methylation in transcription repression, we reasoned that protection of H3K27me2/3 modification by inhibition of corresponding demethylases should repress AR-driven transcription in PCa and CRPC, thus providing a new potential options for PCa treatment (the model in Figure 7). The small molecule inhibitor of JMJD3/UTX was recently suggested as a novel therapy for pediatric brainstem glioma [14] and T-cell acute lymphoblastic leukemia [13]. We analyzed effect of JMJD3/UTX inhibitor GSK-J4 on proliferation of PCa and CRPC cell lines. Our data showed that GSK-J4 effectively reduced proliferation in multiple prostate cancer cell lines, including PCa and CRPC cells, at the micromolar level (Figure 1). Importantly, our results demonstrate that GSK-J4 is more potent in reducing proliferation potential of ARΔLBD CRPC cells compared to isogenic cells expressing AR WT cells. In this direction, we were able to characterize dynamics of H3K27me1 after GSK-J4 treatment and observed much faster reduction of this modification in CRPC cells compared to PCa cells (Figure 4). Minor accumulation H3K27me3 was found only in R1-D567 cells, and no accumulation was observed in R1-AD1 or CWR22Rv-1 cells (Figure 4). These data can be interpreted in the context of the previous report that the inhibition of JMJD3/UTX with GSK-J4 did not induce the global accumulation of H3K27me3, but rather elevated levels of this modification at the specific promoter regions [13]. These data may suggest that inhibition of JMJD3/UTX diminished proliferation of tested CRPC cells by stabilization of H3K27me3 at the AR-induced elements, thus hindering AR-driven transcription; further experiments are necessary to address this hypothesis. In addition, we observed that GSK-J4 has prolonged activity as cells pre-treated with this inhibitor have reduced proliferation in colony formation assay (Figure 3). To understand the mechanism of this long-term activity, it would be necessary to test what is the dynamics of H3K27me1 recovery after inhibitor withdrawal and what are the specific immediate target genes that are affected by the treatment.

AR interacts with, and its transcription activity is regulated by several members of histone demethylase family, including JARID1B (KDM5B) [33], JHDM2A (KDM3A) [34], JMJD2A (KDM4A) and JMJD2D (KDM4D) [35]. Thus, one of the possible mechanisms of JMJD3/UTX-dependent regulation of AR activity could involve an AR-mediated recruitment of JMJD3/UTX complexes to the androgen response elements (ARE) for H3K27 demethylation and transcription activation. In order to address this hypothesis, further studies of potential AR-JMJD3/UTX interaction and AR-dependent recruitment to ARE will be required. In addition, this conceivable interaction may explain an observed difference in inhibition response between R1-AD1 (AR WT) and CRPC cells, which expressed ARΔLBD that has a permanent nuclear localization and, as such, can be more sensitive to the changes in the epigenetic modification. If interaction between AR and JMJD3/UTX is confirmed, it may warrant further studies directed to interfere with this interaction to block demethylation at AREs and to reduce cell proliferation.

Another important question to be addressed is the mode of cell response to GSK-J4 treatment and mechanism of cell death induced by GSK-J4 treatment. At the ED50 concentrations, effect of treatment is mostly cytostatic (Figure 2); yet, cell counting method that was used in this study cannot discriminate between several possibilities that may include complete block of cell cycle progression (that should result in the accumulation of cells in G0) and partial retention of proliferation activity in combination with cell death.

Cabazitaxel, a taxane derivative, is one of the few therapeutic options recently approved for CRPC treatment [20, 22]. Combined treatment of GSK-J4 and Cabazitaxel was tested in order to discover potential drugs synergism and to improve the chemotherapy efficacy. We observed that combined treatment of Cabazitaxel and GSK-J4 reduced proliferation of CRPC cells more effectively compared to PCa cells. Two drugs affect different pathways in cells, and observed synergism can be potentially attributed to the increased sensitivity of GSK-J4 treated cells to the mitotic block that is activated by taxanes, including Cabazitaxel, at the clinically relevant nanomolar concentrations (Figure 5).

In this study, we aimed to investigate the manipulating of histone modification, specifically H3K27 methylation, using the small molecule demethylase inhibitor GSK-J4. Collectively, our data indicated that GSK-J4 is promising to be further developed as a novel treatment approach in CRPC and highlighted JMJD3/UTX as a potential new target in CRPC therapy. In the future studies, it will be necessary to address the balance between methytransferase EZH2 and demethylases JMJD3/UTX in PCa and CRPC cell lines, and, importantly, in the primary patients PCa samples, that can warrant H3K27 methylation pathway as a therapeutic target for CRPC treatment.

## MATERIALS AND METHODS

### Cell culture

R1-AD1, R1-D567 and R1-I567 [16], CWR22Rv-1 and PC3 cells were cultured in RPMI 1640 medium with L-glutamine (Corning #10-040-CV) supplemented with 10% fetal bovine serum (Thermo Fisher Scientific #10437-036) and penicillin/streptomycin (Corning #30-002-Cl) in a humidified incubator at 37°C with 5% CO2. JMJD3/UTX inhibitor GSK-J4 (Sigma #SML0701) and Cabazitaxel (LC Laboratories #C-2581) were dissolved in DMSO (Sigma #D2650) to prepare 20mM and 20μM stock correspondingly.

### Cells viability assays on GSK-J4 treatment

Cells were plated on 12-well plates, 4x10^5^ cell/well. After 24h, GSK-J4 was diluted in the cell culture medium and was added to cells for 72h treatment. Cells were trypsinized, collected in 1mL RPMI and stain with 0.4% Trypan Blue solution, the cell number of each sample was acquired using Countess® II Automated Cell Counter (Thermo Fisher Scientific). Time-course experiment was performed in the same setting as above. Cell number was acquired using Countess® II Automated Cell Counter at time point 24 h, 48 h, 72 h and 96 h after treatment. Samples were triplicated for statistic purpose. Alamar® blue assay was also used for cells viability test after 72 h GSK-J4 treatment. Cells were set 1000 cell/well on 96-well plates; 10 μL Alamar® blue reagent (Thermo Scientific #00-100) was added to each well. Data was collected using Spectra Max M3 plate reader after 6 h signal development.

### Colony formation assay with GSK-J4 treated prostate cancer cells

Cells were treated with GSK-J4 in different concentrations for 72h. Next, cells were trypsinezed and transferred to 6-well plates in two different sets: 1) cells were diluted 1: 10000; 2) cells were stained with Trypan Blue, counted and diluted to 200 viable cells/well. Cells were growing for 10 days until colonies became visible. Formed colonies were fixed with 1% formaldehyde for 10 min, washed with PBS buffer, and stained with 0.5% crystal violet was used to stain colonies for 15 min. Colonies were analyzed using ImageQuant 400 imager. The experiment was performed in triplicate.

### Western blot

Cells were washed with PBS and collected in Laemmli buffer directly. Protein samples were separated by 4-20% SDS-PAGE precast gel (Bio-Rad #456-1096) at 200 V for 30 min, and then transferred to nitrocellulose membranes (Whatman, Dassel, Germany) at 30V for 70 min. Membranes were blocked with 4% non-fat milk in PBS-0.1%Tween buffer (PBST) for 30min at room temperature. Primary antibodies anti-H3K27me3 (Millipore #07-449), anti-H3K27me1 (Millipore #07-448), anti-histone H3 (Abcam #ab1791), anti-JMJD3 (Abcam # ab85392), UTX (Abcam # ab36938), and anti-α-actin (Sigma #A5316) were diluted in 4% non-fat milk in PBST and incubated with membranes overnight at 4°C. Membranes were wash for 3 times with PBST and then incubated with secondary antibodies (Millipore) for 1 h at room temperature. Membranes were then washed with PBST 3 times and visualized using ECL reagent. Western blots analysis was performed using software Image J.

### GSK-J4 - Cabazitaxel combined treatment and synergistic analysis

Cells were plated on 96-well plates with concentration 1000 cells/well. Combined drugs were added after 24 h settlement. The combined drug doses were designed as 0+0(naive background), 0+0 (control with DMSO background), 0.25+0.25, 0.5+0.5, 1+1, 2+2, 4+4, 8+8, 16+16, 32+32 (μM GSK-J4 + nM Cabazitaxel). Cell viability was characterized using Alamar® Blue assay. Synergistic analysis was performed based on Chou and Talalay’s model on multiple drug-effect and the combination index theorem [36] using the software CompuSyn®.

### Cell morphology analysis

Cells were treated with indicated concentrations of Cabazitaxel for 24h. The cell-permeable Hoechst 33342 stain (Sigma) was added directly to cell culture media to final concentration 10 μg/ml for 20 min followed by cell fixation with 1% formaldehyde for 10 min and microscopy documentation. Morphology analysis was done as described previously [25].

## SUPPLEMENTARY MATERIALS FIGURES



## Abbreviations

AR: androgen receptor
CRPC: castration resistant prostate cancer
H3K27Me: histone H3 methylated at lysine 27
JMJD3 (also KDM6B): histone H3K27 demethylase
LBD: ligand-binding domain
PCa: prostate cancer
UTX (also KDM6A): histone H3K27 demethylase

## References

[1] Siegel R, Naishadham D, Jemal A (2013). Cancer statistics, 2013. CA Cancer J Clin.

[2] Rathkopf D, Scher HI (2013). Androgen receptor antagonists in castration-resistant prostate cancer. Cancer J.

[3] Egan A, Dong Y, Zhang H, Qi Y, Balk SP, Sartor O (2014). Castration-resistant prostate cancer: adaptive responses in the androgen axis. Cancer Treat Rev.

[4] Shafi AA, Cox MB, Weigel NL (2013). Androgen receptor splice variants are resistant to inhibitors of Hsp90 and FKBP52, which alter androgen receptor activity and expression. Steroids.

[5] Gottlieb B, Beitel LK, Nadarajah A, Paliouras M, Trifiro M (2012). The androgen receptor gene mutations database: 2012 update. Hum Mutat.

[6] Joseph JD, Lu N, Qian J, Sensintaffar J, Shao G, Brigham D, Moon M, Maneval EC, Chen I, Darimont B, Hager JH (2013). A clinically relevant androgen receptor mutation confers resistance to second-generation antiandrogens enzalutamide and ARN-509. Cancer Discov.

[7] Dehm SM, Tindall DJ (2011). Alternatively spliced androgen receptor variants. Endocr Relat Cancer.

[8] Attard G, Richards J, de Bono JS (2011). New strategies in metastatic prostate cancer: targeting the androgen receptor signaling pathway. Clinical cancer research.

[9] Pellakuru LG, Iwata T, Gurel B, Schultz D, Hicks J, Bethel C, Yegnasubramanian S, De Marzo AM (2012). Global levels of H3K27me3 track with differentiation *in vivo* and are deregulated by MYC in prostate cancer. Am J Pathol.

[10] Deligezer U, Yaman F, Darendeliler E, Dizdar Y, Holdenrieder S, Kovancilar M, Dalay N (2010). Post-treatment circulating plasma BMP6 mRNA and H3K27 methylation levels discriminate metastatic prostate cancer from localized disease. Clin Chim Acta.

[11] Xiang Y, Zhu Z, Han G, Lin H, Xu L, Chen CD (2007). JMJD3 is a histone H3K27 demethylase. Cell Res.

[12] Kruidenier L, Chung CW, Cheng Z, Liddle J, Che K, Joberty G, Bantscheff M, Bountra C, Bridges A, Diallo H, Eberhard D, Hutchinson S, Jones E (2012). A selective jumonji H3K27 demethylase inhibitor modulates the proinflammatory macrophage response. Nature.

[13] Ntziachristos P, Tsirigos A, Welstead GG, Trimarchi T, Bakogianni S, Xu L, Loizou E, Holmfeldt L, Strikoudis A, King B, Mullenders J, Becksfort J, Nedjic J (2014). Contrasting roles of histone 3 lysine 27 demethylases in acute lymphoblastic leukaemia. Nature.

[14] Hashizume R, Andor N, Ihara Y, Lerner R, Gan H, Chen X, Fang D, Huang X, Tom MW, Ngo V, Solomon D, Mueller S, Paris PL (2014). Pharmacologic inhibition of histone demethylation as a therapy for pediatric brainstem glioma. Nat Med.

[15] Li Y, Hwang TH, Oseth LA, Hauge A, Vessella RL, Schmechel SC, Hirsch B, Beckman KB, Silverstein KA, Dehm SM (2012). AR intragenic deletions linked to androgen receptor splice variant expression and activity in models of prostate cancer progression. Oncogene.

[16] Nyquist MD, Li Y, Hwang TH, Manlove LS, Vessella RL, Silverstein KA, Voytas DF, Dehm SM (2013). TALEN-engineered AR gene rearrangements reveal endocrine uncoupling of androgen receptor in prostate cancer. Proc Natl Acad Sci USA.

[17] Park SY, Park JW, Chun YS (2016). Jumonji histone demethylases as emerging therapeutic targets. Pharmacol Res.

[18] Jordan MA, Wilson L (2004). Microtubules as a target for anticancer drugs. Nat Rev Cancer.

[19] Dumontet C, Jordan MA (2010). Microtubule-binding agents: a dynamic field of cancer therapeutics. Nat Rev Drug Discov.

[20] Abidi A (2013). Cabazitaxel: A novel taxane for metastatic castration-resistant prostate cancer-current implications and future prospects. J Pharmacol Pharmacother.

[21] de Bono JS, Oudard S, Ozguroglu M, Hansen S, Machiels JP, Kocak I, Gravis G, Bodrogi I, Mackenzie MJ, Shen L, Roessner M, Gupta S, Sartor AO, TROPIC Investigators (2010). Prednisone plus cabazitaxel or mitoxantrone for metastatic castration-resistant prostate cancer progressing after docetaxel treatment: a randomised open-label trial. Lancet.

[22] de Leeuw R, Berman-Booty LD, Schiewer MJ, Ciment SJ, Den RB, Dicker AP, Kelly WK, Trabulsi EJ, Lallas CD, Gomella LG, Knudsen KE (2015). Novel actions of next-generation taxanes benefit advanced stages of prostate cancer. Clin Cancer Res.

[23] Musacchio A, Salmon ED (2007). The spindle-assembly checkpoint in space and time. Nat Rev Mol Cell Biol.

[24] Giovinazzi S, Bellapu D, Morozov VM, Ishov AM (2013). Targeting mitotic exit with hyperthermia or APC/C inhibition to increase paclitaxel efficacy. Cell Cycle.

[25] Lindsay CR, Scholz A, Morozov VM, Ishov AM (2007). Daxx shortens mitotic arrest caused by paclitaxel. Cell Cycle.

[26] Wertz IE, Kusam S, Lam C, Okamoto T, Sandoval W, Anderson DJ, Helgason E, Ernst JA, Eby M, Liu J, Belmont LD, Kaminker JS, O’Rourke KM (2011). Sensitivity to antitubulin chemotherapeutics is regulated by MCL1 and FBW7. Nature.

[27] Vaishampayan U (2014). Therapeutic options and multifaceted treatment paradigms in metastatic castrate-resistant prostate cancer. Curr Opin Oncol.

[28] Smith DC, Smith MR, Sweeney C, Elfiky AA, Logothetis C, Corn PG, Vogelzang NJ, Small EJ, Harzstark AL, Gordon MS, Vaishampayan UN, Haas NB, Spira AI (2013). Cabozantinib in patients with advanced prostate cancer: results of a phase II randomized discontinuation trial. J Clin Oncol.

[29] Patel JC, Maughan BL, Agarwal AM, Batten JA, Zhang TY, Agarwal N (2013). Emerging molecularly targeted therapies in castration refractory prostate cancer. Prostate Cancer.

[30] Slovin SF, Higano CS, Hamid O, Tejwani S, Harzstark A, Alumkal JJ, Scher HI, Chin K, Gagnier P, McHenry MB, Beer TM (2013). Ipilimumab alone or in combination with radiotherapy in metastatic castration-resistant prostate cancer: results from an open-label, multicenter phase I/II study. Ann Oncol.

[31] Kantoff PW, Schuetz TJ, Blumenstein BA, Glode LM, Bilhartz DL, Wyand M, Manson K, Panicali DL, Laus R, Schlom J, Dahut WL, Arlen PM, Gulley JL, Godfrey WR (2010). Overall survival analysis of a phase II randomized controlled trial of a Poxviral-based PSA-targeted immunotherapy in metastatic castration-resistant prostate cancer. J Clin Oncol.

[32] Duan L, Rai G, Roggero C, Zhang QJ, Wei Q, Ma SH, Zhou Y, Santoyo J, Martinez ED, Xiao G, Raj GV, Jadhav A, Simeonov A (2015). KDM4/JMJD2 Histone Demethylase Inhibitors Block Prostate Tumor Growth by Suppressing the Expression of AR and BMYB-Regulated Genes. Chem Biol.

[33] Xiang Y, Zhu Z, Han G, Ye X, Xu B, Peng Z, Ma Y, Yu Y, Lin H, Chen AP, Chen CD (2007). JARID1B is a histone H3 lysine 4 demethylase up-regulated in prostate cancer. Proc Natl Acad Sci USA.

[34] Yamane K, Toumazou C, Tsukada Y, Erdjument-Bromage H, Tempst P, Wong J, Zhang Y (2006). JHDM2A, a JmjC-containing H3K9 demethylase, facilitates transcription activation by androgen receptor. Cell.

[35] Shin S, Janknecht R (2007). Activation of androgen receptor by histone demethylases JMJD2A and JMJD2D. Biochem Biophys Res Commun.

[36] Chou TC (2006). Theoretical basis, experimental design, and computerized simulation of synergism and antagonism in drug combination studies. Pharmacol Rev.

