# Supplementary material for: Inhibitor of H3K27 demethylase JMJD3/UTX GSK-J4 is a potential therapeutic option for castration resistant prostate cancer
Source: Oncotarget. 2017 Jul 8;8(37):62131–42. doi: 10.18632/oncotarget.19100 (PMC5617492; doi:10.18632/oncotarget.19100)
Supplement: Supplementary file 1 [file oncotarget-08-62131-s001.pdf]

# Inhibitor of H3K27 demethylase JMJD3/UTX GSK-J4 is a potential therapeutic option for castration resistant prostate cancer

## SUPPLEMENTARY MATERIALS

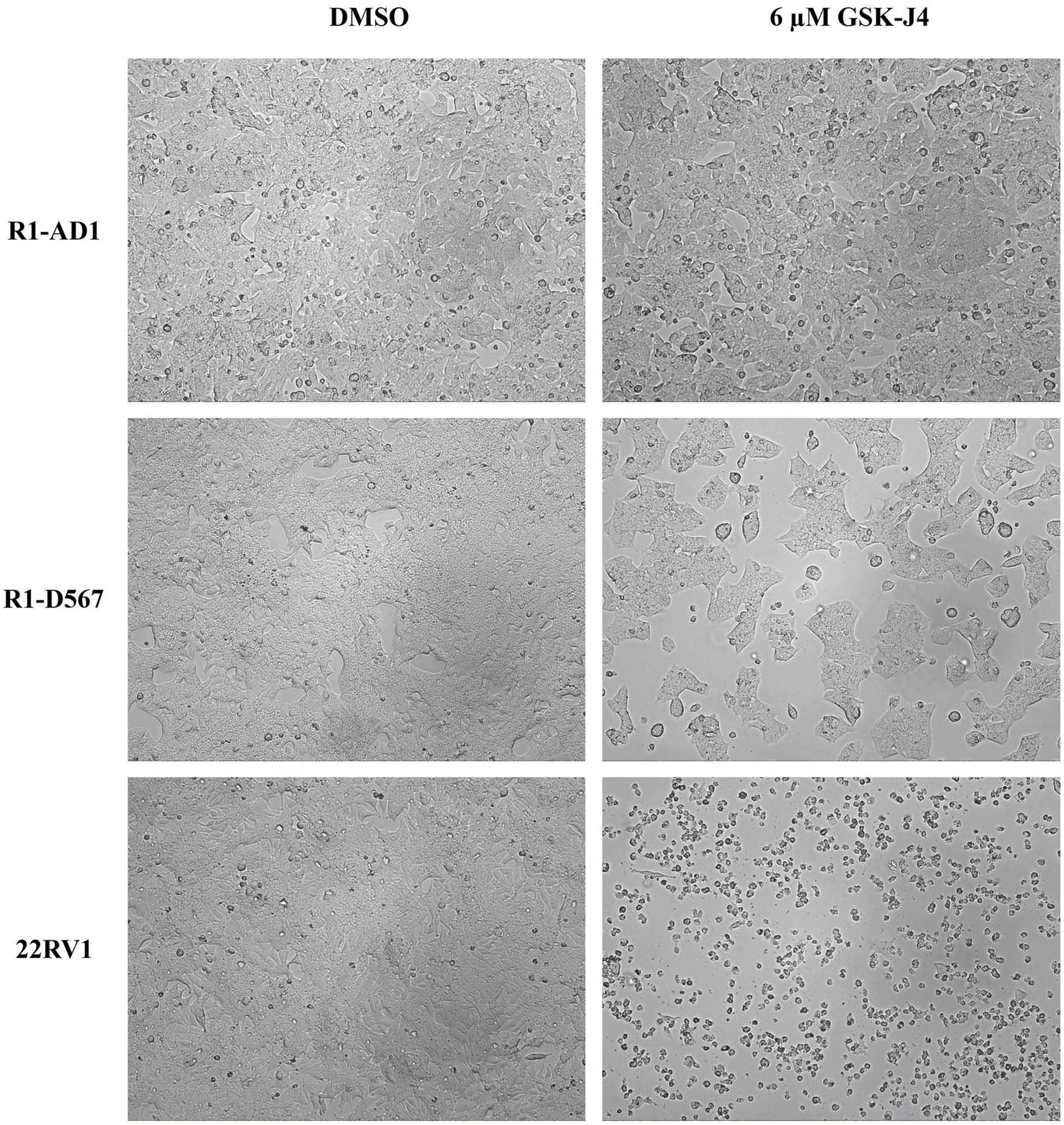


**Supplementary Figure 1: GSK-J4 treatment represses PCa cells proliferation.** Analysis of cell morphology in control-treated or GSK-J4-treated (6µM for 72h) cells.

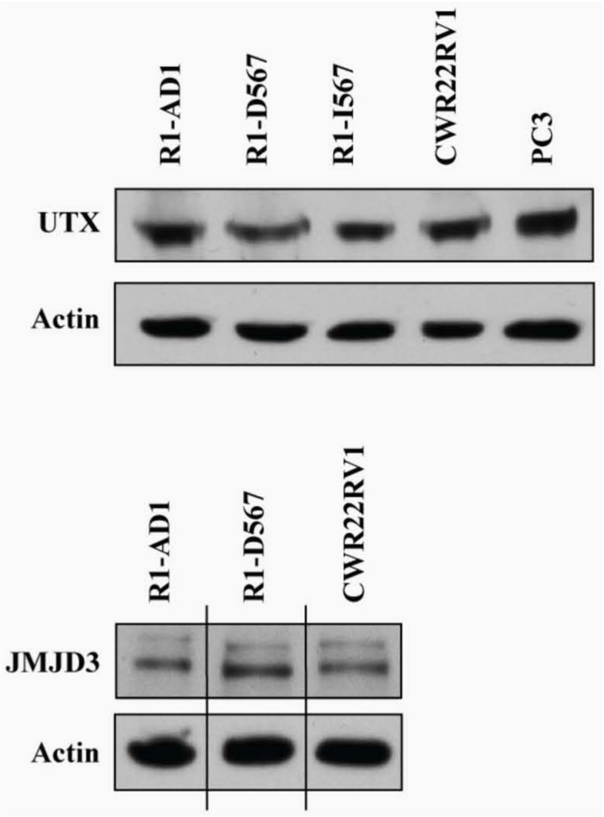


**Supplementary Figure 2: Analysis of JMJD3 and UTX levels.** Western blot analysis of JMJD3 and UTX. Beta-actin for loading normalization.

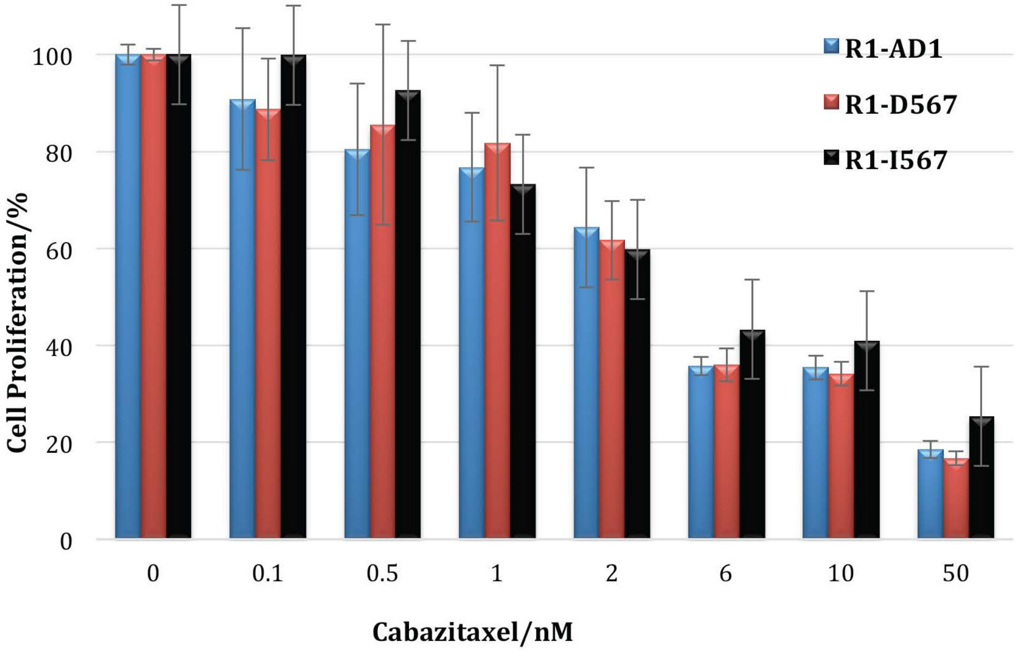


**Supplementary Figure 3: Proliferation of cells treated with Cabazitaxel.** ED50 of Cabazitaxel was determined for R1-AD1, R1-D567 and R1-I567 cells.
